# Spatiotemporal distribution and risk factors for patient and diagnostic delays among groups with tuberculous pleurisy: an analysis of 5-year surveillance data in eastern China

**DOI:** 10.3389/fpubh.2024.1461854

**Published:** 2024-09-09

**Authors:** Yang Li, Dan Luo, Yi Zheng, Kui Liu, Songhua Chen, Yu Zhang, Wei Wang, Qian Wu, Yuxiao Ling, Yiqing Zhou, Bin Chen, Jianmin Jiang

**Affiliations:** ^1^School of Public Health, Hangzhou Normal University, Hangzhou, Zhejiang, China; ^2^School of Public Health, Hangzhou Medical College, Hangzhou, Zhejiang, China; ^3^Department of Tuberculosis Control and Prevention, Jiaxing Nanhu District Center for Disease Control and Prevention, Jiaxing, Zhejiang, China; ^4^Department of Tuberculosis Control and Prevention, Zhejiang Provincial Center for Disease Control and Prevention, Hangzhou, Zhejiang, China; ^5^School of Public Health, Health Science Center, Ningbo University, Ningbo, Zhejiang, China; ^6^Key Laboratory of Vaccine, Prevention and Control of Infectious Disease of Zhejiang Province, Zhejiang Provincial Center for Disease Control and Prevention, Hangzhou, Zhejiang, China

**Keywords:** tuberculous pleurisy, patient delay, diagnostic delay, spatiotemporal distribution, risk factors

## Abstract

**Objective:**

To understand and analyze the factors relating to patient and diagnostic delays among groups with tuberculous pleurisy (TP), and its spatiotemporal distribution in Zhejiang Province.

**Methods:**

Data of all tuberculous pleurisy patients were collected from the existing Tuberculosis Information Management System. A time interval of > 2 weeks between first symptom onset and visit to the designated hospital was considered a patient delay, and a time interval of > 2 weeks between the first visit and a confirmed TP diagnosis was considered a diagnostic delay. Univariate and multivariate logistic regression analyses were used to explore factors influencing patient and diagnostic delays in patients with TP. Spatial autocorrelation and spatiotemporal scan analyses were used to identify hot spots and risk clusters, respectively.

**Results:**

In total, 10,044 patients with TP were included. The median time and interquartile range for patients seeking medical care and diagnosis were 15 (7–30) and 1 (0–8) days, respectively. The results showed that people aged > 65 years, retirees, and residents of Jinhua, Lishui, and Quzhou were positively correlated with patient delay, whereas retreatment patients, houseworkers, unemployed people, and residents of Zhoushan or Ningbo were positively correlated with diagnostic delay. Additionally, high-risk clusters of patient delays were observed in the midwestern Zhejiang Province. The most likely clusters of TP diagnostic delays were found in southeast Zhejiang Province.

**Conclusion:**

In summary, patient delay of TP in Zhejiang province was shorter than for pulmonary tuberculosis in China, while the diagnostic delay had no difference. Age, city, occupation, and treatment history were related to both patient and diagnostic delays in TP. Interventions in central and western regions of Zhejiang Province should be initiated to improve the early detection of TP. Additionally, the allocation of health resources and accessibility of health services should be improved in the central and eastern regions of Zhejiang Province.

## 1 Introduction

Tuberculosis (TB) is a chronic infectious disease caused by *Mycobacterium tuberculosis*. Based on the latest global TB report, the estimated number of patients with TB worldwide in 2022 is 10.6 million, in which the incidence of TB (new cases per 100,000 population per year) is estimated to increase by 3.9% between 2020 and 2022 (1). TB generally targets the lungs but can also affect other sites, such as the pleura (2). China is one of the countries with the highest burden, accounting for 7.1% of the global total, and tuberculous pleurisy (TP) is a subtype of TB found in China (1). The incidence of TP in China is on the rise, from 0.4 per 100,000 people in 2005 to 3.3 per 100,000 people in 2018, and has attracted widespread attention (3).

Tuberculous pleurisy presents clinically as a pleural effusion with pain and fever, affecting breathing and eventually leading to a long-term impairment of lung function (4). Owing to a slow course and lack of specificity in symptoms and identification, TP is difficult to diagnose and prone to various delays (5). These delays include patient delays and diagnostic delays. The patient delay will not only lead to worsening the patient’s condition, setting back the best opportunity for early diagnosis and treatment, but also greatly increasing the risk of infection in the community (6). In addition, the diagnostic delay will lead to delays in treatment (7). Spatial epidemiology has been widely used to describe the distribution and clustering of diseases in time and space (8). Compared to traditional epidemiological techniques, spatiotemporal analysis has shown important advantages in the surveillance of infectious diseases, quantification of susceptible populations, and effective allocation of health resources (9). The results of spatiotemporal analysis can help control an epidemic rapidly, formulate corresponding health policies, and optimize health resources. In this study, we chose the Zhejiang Province, which is an economically developed area in coastal China with a moderate TB burden.

This study aimed to analyze the patient and diagnostic delays of TP, including determining the epidemiological characteristics, indentifing the influencing factors, and finding the spatiotemporal distribution of TP in Zhejiang Province. This study will provide innovative ideas for the prevention and control of epidemics and promote the optimization of current health policies to end TB.

## 2 Materials and methods

### 2.1 Overview of the study area

Zhejiang Province, located on the east coast of China, covers an area of 101,800 km^2^, accounting for 1.06% of the national land area (10). The province includes plains, mountains, coasts, islands, and lakes (10). There are two sub-provincial cities and nine prefecture-level cities in Zhejiang Province (11). In addition, according to the latest data released by the Zhejiang Provincial Bureau of Statistics, the province’s permanent population was 66.27 million at the end of 2023 (12).

### 2.2 Data source and definition

All TP cases identified in Zhejiang province between 2019 and 2023 were obtained from the Tuberculosis Information Management System (TBIMS), which collected demographic and clinical data of all the patients during the specified period. The diagnostic criteria for TP used in this study were the criteria set forth by the National Health and Family Planning Commission in 2017 (WS288-2017) (13) and the Chinese Tuberculosis Prevention and Control Technical Guidelines. the clinical diagnostic criteria for tuberculous pleurisy are as follows: Chest imaging shows lesions consistent with active pulmonary tuberculosis, and other pulmonary diseases are excluded through differential diagnosis. Diagnosis of tuberculous pleurisy can be made if pleural effusion is identified as exudate, adenosine deaminase (ADA) is elevated, and any of the followings such as moderate or higher positive tuberculin test, positive interferon-gamma release assay, or positive anti-tuberculosis antibody test. Besides, the laboratory diagnostic criteria for tuberculous pleurisy require meeting at least one of the following items: (1) Pleural fluid or pleural pathology examination mainly shows epithelioid granulomatous inflammation. (2) Pathogen examination of pleural fluid shows bacteriological examination positive for smear microscopy; positive Mycobacterium culture with the strain identified as *Mycobacterium tuberculosis* complex; or molecular biological examination positive for *Mycobacterium tuberculosis* nucleic acid.

The criteria for delay were as follows: patient delay was defined as the interval between first symptom onset and visit to the designated hospital of > 2 weeks, while a diagnostic delay was defined as the interval between the first visit and a confirmed TP diagnosis of > 2 weeks (14).

### 2.3 Epidemiological characteristics analysis

General epidemiological characteristics were analyzed using R software (version 4.3.0; R Core Team, Vienna, Austria). The influencing factors were analyzed using SPSS software (version 26.0). Variables with a *p-*value of <0.1 in univariate logistic regression analysis were included in multivariate analysis.

### 2.4 Spatial autocorrelation analysis

Global and local spatial autocorrelation analyses evaluated the correlation of variables based on their spatial location similarity to reveal the spatial distribution of diseases (15). Among them, global autocorrelation compared the attributed values aggregated in the whole region with those in each county unit to obtain the average degree of correlation between patients with TP and diagnostic delays in each county to determine whether there was spatial heterogeneity in delays in the diagnosis of TP across the province (16). The global value of Moran’s I ranges from −1 to 1. The closer Moran’s I is to 1, the more clustered the values of adjacent regions are, and the stronger the positive spatial autocorrelation is. The closer Moran’s I is to −1, the higher and lower values in the space are interspersed, and Moran’s *I* = 0 indicates no spatial heterogeneity in the global scope, and the data are randomly distributed (15). When *p* < 0.05 and the *Z-*value ≥1.96, Moran’s *I* difference was considered statistically significant.

In addition, local indicators of spatial autocorrelation (LISA) compiled by partial Moran’s *I* can be used to determine the correlation between county and district variables (17). “Low-High” indicates that the low-incidence area is surrounded by the high-incidence area; “High-Low” indicates that the high-incidence area is surrounded by the low-incidence area; “High-High” indicates the hot spot; “Low-Low” indicates the cold spot; and “not significant” means no spatial autocorrelation (17). Local spatial autocorrelation identifies statistically significant high-prevalence areas (hot spots) and low-prevalence areas (cold spots) of TP delay in Zhejiang Province by detecting spatial autocorrelation between the study variable and the adjacent regional variables. The above analysis was performed using Geoda (version 1.20).

### 2.5 Spatiotemporal scan statistics

Kulldorff spatiotemporal scanning statistics were used to conduct monthly spatiotemporal analysis of the data from different counties and districts, and the spatial units and time of potential clusters of variables were identified. The rates of patient and diagnostic delays in patients with TP in these potential clusters were significantly higher than those in nearby areas (*p* < 0.05) (15). Kulldorff spatiotemporal scanning statistics are based on a mobile cylindrical window (two-dimensional) containing geographical information. The radius corresponds to the space covered by the bottom-scanning window and the height corresponds to time. All spatial units were scanned with changes in radius and height, and the incidences of delay inside and outside the window were compared (18). The null hypothesis was that the relative risk (RR) of patient/diagnosis delay within the window was equal to the relative risk outside the window. Patients with TP within the cluster were more likely to have a patient/diagnosis delay than those outside the cluster when RR > 1, whereas when RR < 1, the cluster was protective (19). The spatiotemporal scanning window with the largest log-likelihood ratio (LLR) was called the most likely cluster, and the other windows were called secondary clusters. We used Monte Carlo simulations to test the significance of the identified clusters at a 95% confidence level (CI) (20). The LLR and RR were calculated as follows:


(1) $$LLR=\log{\left(\frac{c}{E\left[c\right]}\right)}^{c}{\left(\frac{C-c}{C-E\left[c\right]}\right)}^{C-c}\cdot I\left(\right)$$

(2) $$RR=\frac{c/E\left[c\right]}{\left(C-c\right)/\left(C-E\left[c\right]\right)}$$

In equations (1) and (2) C is the total number of patient delays or diagnosis delays in patients with TP, c is the number of delays observed within the window, $E\left[c\right]$ equal to the expected number of delays within the window after covariate adjustment under the null hypothesis, $C-E\left[c\right]$ is the expected number of delays outside the window, and $I\left(\right)$ is the indicator function (21). Spatiotemporal analyses were performed using SaTScan (version 10.1.1, Boston, MA, United States) and visualized using ArcGIS software (version 10.8, SERI Inc.; Redlands, CA, United States).

## 3 Results

### 3.1 General epidemiological features

Between 2019 and 2023, 10,044 cases of TP were identified in Zhejiang Province in the TBIMS. Among them, 6,827 were males and 3,217 were females, with a male-to-female ratio of 2:1. The time interval to seeking medical care and diagnosis was 15 (interquartile range [IOR]: 7–30) and 1 (IQR: 0–8) days, respectively. The age distribution showed that the time interval to seek medical care in the 0–14, 15–64, and 65 and older age groups was 12.5 (IQR: 5.8–30), 14 (IQR: 6–30), and 17 (IQR: 8–30) days, respectively, and the time interval to diagnosis was slightly longer in patients aged 0–14 years at 2 (IQR: 0–8) days. Among different occupations, agricultural workers had the highest number of patient and diagnostic delays. According to the city, the time interval for seeking medical care was the longest in Jinhua (21 [IQR: 11–30] days), and the time to diagnosis was the longest in Zhoushan (8 [IQR: 0–8] days). Regarding case findings, the time interval to seek medical care for active detection cases was slightly longer than that for passive detection cases at 16 (IQR: 7.5–30) days. Regarding treatment history, the time interval to diagnosis for initial treatment and retreatment were 1 (IQR: 0–8) and 2 (IQR: 0–8) days, respectively. Patients who had received anti-TB treatment waited longer to seek medical care (15 days) than those who had not previously been treated for TB (Table 1).

**Table 1:** General epidemic characteristics of patients with TP in Zhejiang Province between 2019 and 2023.

Characteristics	Total	Seeking medical care		Diagnosis	
		Median (IQR)	Cases of patient delay	Median (IQR)	Cases of diagnosis delay
Total PTB	10,044 (100)	15 (7–30)	5,176 (100)	1 (0–8)	1,343 (100)
Sex					
Male	6,827 (68.8)	15 (7–30)	3,543 (68.5)	1 (0–8)	906 (67.5)
Female	3,217 (32.0)	15 (7–30)	1,633 (31.5)	1 (0–8)	437 (32.5)
Age group (years)					
0–14	68 (0.7)	12.5 (5.8–30)	24 (0.5)	2 (0–8)	7 (0.5)
15–64	6,262 (62.3)	14 (6–30)	3,052 (59.0)	1 (0–8)	830 (61.8)
65 and above	3,714 (37.0)	17 (8–30)	2,100 (40.6)	1 (0–8)	506 (37.7)
Occupation					
Industrial workers	830 (8.3)	12 (5–30)	372 (7.2)	1 (0–8)	118 (8.8)
Agriculture workers	5,272 (52.8)	16 (7–30)	2,833 (54.7)	1 (0–8)	632 (47.1)
Houseworkers or unemployed	1,257 (12.5)	13 (5–30)	586 (11.3)	2 (0–8)	249 (18.5)
Retiree	733 (7.3)	17 (9–30)	426 (8.2)	2 (0–8)	108 (8.0)
Commercial service stratum	526 (5.2)	12.5 (5.3–30)	243 (4.7)	3 (0–8)	75 (5.6)
Students	377 (3.4)	13 (5–30)	137 (2.6)	1 (0–8)	42 (3.1)
Others	1,089 (10.8)	15 (7–30)	579 (11.2)	1 (0–8)	119 (8.9)
City					
Hangzhou	1,894 (18.9)	12 (5–30)	835 (16.1)	3 (0–8)	265 (19.7)
Ningbo	1,193 (11.9)	14 (6–30)	565 (10.9)	5 (0–8)	235 (17.5)
Wenzhou	1,152 (11.5)	15 (4–30)	615 (11.9)	0 (0–8)	185 (13.8)
Jiaxing	711 (7.1)	13 (6–30)	323 (6.2)	0 (0–8)	81 (6.0)
Huzhou	571 (5.7)	12 (2–30)	244 (4.7)	0 (0–8)	64 (4.8)
Shaoxing	846 (8.4)	18 (8–30)	456 (8.8)	0 (0–8)	73 (5.4)
Jinhua	1,210 (12.0)	21 (11–30)	797 (15.4)	1 (0–8)	131 (9.8)
Quzhou	678 (6.8)	16 (9–30)	379 (7.3)	2 (0–8)	46 (3.4)
Zhoushan	84 (0.8)	13 (6–30)	38 (0.7)	8 (0–8)	30 (2.2)
Taizhou	1,138 (11.3)	15 (7–30)	597 (11.5)	1 (0–8)	171 (12.7)
Lishui	567 (5.6)	17 (8–30)	327 (6.3)	1 (0–8)	62 (4.6)
Case finding					
Passive	10,005 (99.6)	15 (7–30)	5,153 (99.6)	1 (0–8)	1,337 (99.6)
Active	39 (0.4)	16 (7.5–30)	23 (0.4)	1 (0–8)	6 (0.4)
Treatment history					
Initial treatment	9,866 (98.2)	15 (7–30)	5,086 (98.3)	1 (0–8)	1,308 (97.4)
Retreatment	178 (1.8)	15 (3–30)	90 (1.7)	2 (0–8)	35 (2.6)
Anti-TB treatment					
Yes	10,032 (99.9)	15 (7–30)	5,172 (99.9)	1 (0–8)	1,342 (99.9)
No	12 (0.1)	12 (7–30)	4 (0.1)	6 (0–8)	1 (0.1)

### 3.2 Factors associated with the delay

Univariate analysis of demographic characteristics showed that age, occupation, and city were significantly associated with patient delay, while city, occupation, and treatment history were significantly associated with diagnostic delay. Patient and diagnostic delays were used as dependent variables and were included in the multivariate logistic regression analysis.

Multivariate logistic regression analysis showed that the patients aged >65 years were 1.971 times more likely to experience a patient delay than those aged 0–14 years (OR = 1.971, 95% CI: 1.124–3.455). The probability of a patient delay in Jinhua (OR = 2.608, 95% CI: 2.236–3.042), Lishui (OR = 1.812, 95% CI: 1.492–2.199), and Quzhou (OR = 1.598, 95% CI: 1.334–1.914) was 2.608, 1.812, and 1.598 times of that in Hangzhou area, respectively. Additionally, retirees were 1.502 times more likely to experience a patient delay than industrial workers (OR = 1.502, 95% CI: 1.280–1.866).

In addition, the probability of a diagnostic delay in Zhoushan (OR = 3.091, 95% CI: 1.924–4.965) and Ningbo (OR = 1.407, 95% CI: 1.150–1.721) was 3.091 and 1.407 times that of Hangzhou, respectively. The probability of diagnostic delay in retreatment patients (OR = 1.558, 95% CI: 1.066–2.278) was 1.558 times higher than that of patients receiving initial treatment. Moreover, houseworkers or unemployed were 1.361 times more likely to experience a diagnostic delay than industrial workers (OR = 1.361, 95% CI: 1.067–1.736) (Table 2).

**Table 2:** Analysis of influencing factors of patient and diagnosis delays in TP cases in Zhejiang Province between 2019 and 2023.

Characteristic	Patient delay				Diagnosis delay			
	Univariable		Multivariable		Univariable		Multivariable	
	OR (95% CI)	*P-*value	OR (95% CI)	*P-*value	OR (95% CI)	*P-*value	OR (95% CI)	*P-*value
Sex								
Male	Reference	Reference			Reference	Reference		
Female	0.956 (0.879–1.039)	0.288			1.027 (0.909–1.161)	0.667		
Age group (years)								
0–14	Reference	Reference	Reference	Reference	Reference	Reference		
15–64	1.743 (1.057–2.873)	0.029	1.506 (0.866–2.618)	0.147	1.332 (0.607–2.921)	0.475		
65 and above	2.385 (1.445–3.939)	0.001	1.971 (1.124–3.455)	0.018	1.375 (0.625–3.022)	0.429		
Occupation								
Industrial workers	Reference	Reference	Reference	Reference	Reference	Reference	Reference	Reference
Agriculture workers	1.430 (1.234–1.657)	<0.001	1.119 (0.956–1.310)	0.161	0.822 (0.665–1.016)	0.069	0.926 (0.746–1.151)	0.491
Houseworkers or unemployed	1.075 (0.902–1.282)	0.419	0.965 (0.806–1.156)	0.701	1.491 (1.173–1.893)	0.001	1.361 (1.067–1.736)	0.013
Retiree	1.708 (1.398–2.088)	<0.001	1.502 (1.280–1.866)	<0.001	1.043 (0.786–1.383)	0.772	1.068 (0.801–1.425)	0.652
Commercial service stratum	1.057 (0.849–1.316)	0.619	1.117 (0.892–1.399)	0.334	1.003 (0.734–1.372)	0.983	1.024 (0.745–1.409)	0.883
Students	0.843 (0.652–1.090)	0.194	0.814 (0.616–1.075)	0.147	0.859 (0.589–1.253)	0.43	0.903 (0.617–1.321)	0.598
Others	1.398 (1.166–1.676)	<0.001	1.187 (0.987–1.428)	0.069	0.740 (0.564–0.972)	0.03	0.726 (0.551–0.957)	0.023
City								
Hangzhou	Reference	Reference	Reference	Reference	Reference	Reference	Reference	Reference
Ningbo	1.141 (0.987–1.320)	0.075	1.255 (1.079–1.460)	0.003	1.508 (1.243–1.829)	<0.001	1.407 (1.150–1.721)	0.001
Wenzhou	1.452 (1.254–1.683)	<0.001	1.638 (1.403–1.912)	<0.001	1.176 (0.959–1.442)	0.119	1.116 (0.900–1.383)	0.318
Jiaxing	1.056 (0.888–1.256)	0.539	1.138 (0.952–1.359)	0.156	0.790 (0.606–1.030)	0.082	0.729 (0.556–0.956)	0.022
Huzhou	0.946 (0.783–1.143)	0.567	0.992 (0.818–1.202)	0.931	0.776 (0.580–1.038)	0.087	0.743 (0.554–0.997)	0.048
Shaoxing	1.483 (1.260–1.745)	<0.001	1.541 (1.305–1.820)	<0.001	0.581 (0.442–0.763)	<0.001	0.595 (0.451–0.785)	<0.001
Jinhua	2.447 (2.108–2.842)	<0.001	2.608 (2.236–3.042)	<0.001	0.746 (0.597–0.933)	0.01	0.788 (0.627–0.990)	0.041
Quzhou	1.608 (1.347–1.918)	<0.001	1.598 (1.334–1.914)	<0.001	0.447 (0.323–0.620)	<0.001	0.457 (0.329–0.636)	<0.001
Zhoushan	1.048 (0.675–1.625)	0.835	1.122 (0.719–1.751)	0.613	3.415 (2.146–5.436)	<0.001	3.091 (1.924–4.965)	<0.001
Taizhou	1.400 (1.208–1.622)	<0.001	1.497 (1.286–1.744)	<0.001	1.087 (0.883–1.338)	0.432	1.100 (0.887–1.363)	0.385
Lishui	1.728 (1.429–2.089)	<0.001	1.812 (1.492–2.199)	<0.001	0.755 (0.562–1.013)	0.061	0.765 (0.567–1.031)	0.079
Case finding								
Passive	Reference	Reference			Reference	Reference		
Active	1.354 (0.714–2.565)	0.353			1.179 (0.493–2.819)	0.712		
Treatment history								
Initial treatment	Reference	Reference			Reference	Reference	Reference	Reference
Retreatment	0.961 (0.715–1.293)	0.794			1.601 (1.102–2.328)	0.014	1.558 (1.066–2.278)	0.022
Anti-TB treatment								
Yes	Reference	Reference			Reference	Reference		
No	0.470 (0.141–1.561)	0.218			0.589 (0.076–4.563)	0.612		

### 3.3 Spatial autocorrelation analysis

**Figure 1:**
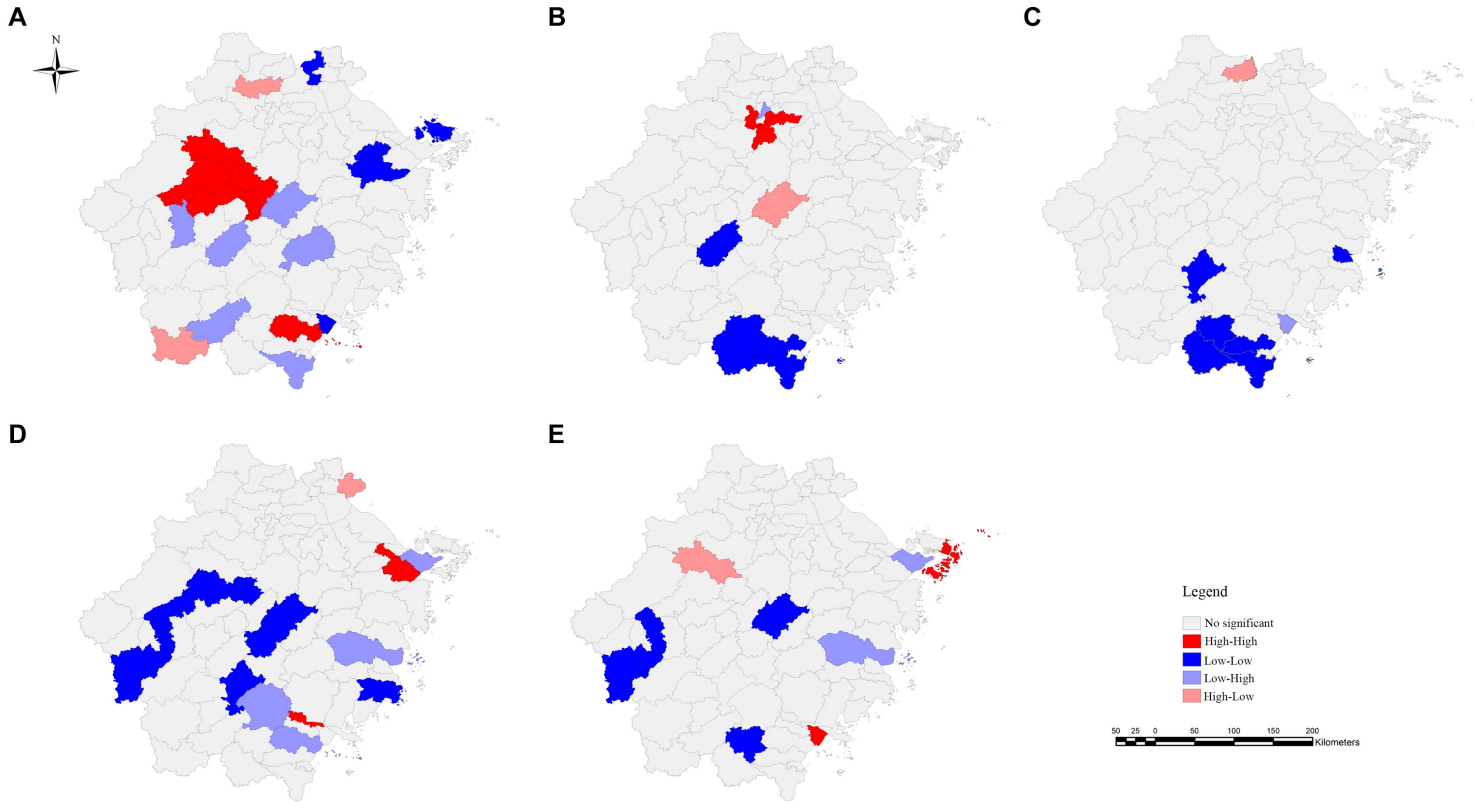
Local indicators of spatial autocorrelation (LISA) plot of TP in Zhejiang Province between 2019 and 2023. Panel **(A)** represents the patient delay in 2019. Panels **(B–D)** represent the diagnostic delays in 2019, 2020, and 2021, respectively. Panel **(E)** represents the diagnostic delay for the period 2019–2022.

Between 2019 and 2023, spatial heterogeneity was found in the patient delay of TP in Zhejiang province only in 2022, and the global Moran’s I was 0.154 (*p* < 0.05). The LISA map showed hot spots located in some counties of Hangzhou (Jiande and Tonglu), Jinhua (Yiwu, Lanxi, and Pujiang), and Wenzhou (Ruian) (Table 3 and Figure 1).

**Table 3:** Global spatial autocorrelation results of TP in Zhejiang Province, 2019–2023.

Year	Patient delay			Diagnosis delay		
	Moran’s *I*	*Z*-value	*p*-value	Moran’s *I*	*Z*-value	*P*-value
2019	−0.087	−1.093	0.139	0.218	3.436	0.005
2020	0.091	1.393	0.081	0.110	1.765	0.038
2021	0.034	0.614	0.278	0.130	1.958	0.038
2022	0.154	2.334	0.016	0.040	0.762	0.210
2023	0.025	0.569	0.270	0.050	0.910	0.182
2019–2023	0.072	1.146	0.129	0.197	3.012	0.005

Spatial heterogeneity was observed in the diagnostic delay of TP in 2019, 2020, and 2021 and within the 5-year range. Moran’s index statistics ranged from 0.110 to 0.218 (*p* < 0.05). The LISA map showed that diagnostic delay clustering occurred in some counties of Hangzhou (Xihu and Xiaoshan), Ningbo (Yinzhou and Jiangbei), Wenzhou (Lucheng and Longwan), and Zhoushan City (Putuo).

### 3.4 Spatiotemporal scan statistics

**Figure 2:**
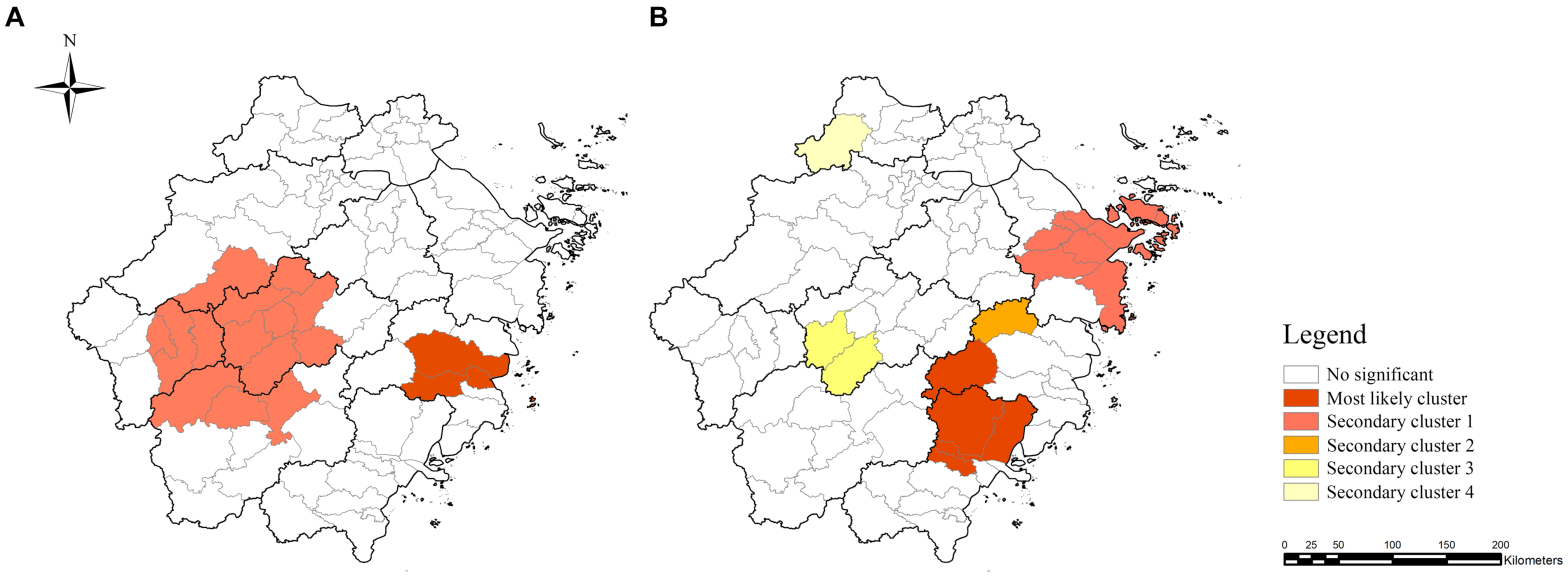
Spatiotemporal clusters of TP in Zhejiang Province between 2019 and 2023. Panels **(A,B)** represent the spatiotemporal clustering of patient and diagnostic delays in TP, respectively.

The statistical results of spatiotemporal scanning, which included monthly time variables, were slightly similar to those of the simple spatial level. In the patient delay of TP in Zhejiang Province between 2019 and 2023, a most likely cluster and a secondary cluster were found. Among them, the most likely clustering time was between January 2019 and September 2021 and was mainly located at the junctional area of the cities of Hangzhou, Quzhou, Jinhua, and Lishui. The counties included Jiande, Kecheng, Qujiang, Longyou, Wucheng, Jindong, Yiwu, Yongkang, Lanxi, Wuyi, Pujiang, Liandu, Suichang, and Songyang (LLR = 30.5; RR = 1.4; *p* < 0.05; Supplementary Table S1-2 and Figure 2).

One most likely cluster and four minor clusters of diagnostic delay were observed, mainly located in the east-central region of Zhejiang Province. The most likely cluster included some counties of Wenzhou (Lucheng, Ouhai, Yueqing, and Yongjia) and Taizhou (Xianju), and the cluster period was between June 2019 and November 2021 (LLR = 48.2; RR = 3.1; *p* < 0.05) (Supplementary Table S1-3 and Figure 2).

## 4 Discussion

Tuberculous pleurisy is one of the most common types of TB and has attracted increasing attention in recent years. Patients with TP occupy a certain proportion of the population in the eastern coastal area of China. Considering a lack of specificity in symptoms and diagnostic methods, diagnosing TP is more difficult than pulmonary tuberculosis (PTB) (5, 22, 23). Additionally, the epidemiological characteristics of TP are not well understood, and there is some spatial heterogeneity in the distribution of delay in TP. Therefore, understanding the delay in patients with TP and analyzing their epidemiological characteristics at the spatiotemporal level is essential.

In this study, males were more likely to develop TP than females, which is consistent with previous findings (24, 25). Considering that clinically TP is secondary to TB, we speculate that the sex difference may be due to differences in risk exposure in daily life (26). In China, most men bear the main economic burden of the family, especially in high-epidemic areas (10, 19). It could also be that the underlying genetic and hormonal mechanisms differ between males and females (27). Available evidence suggests that sex hormones and genetic effectors encoded on sex chromosomes may be determinants of immunity and disease susceptibility that influence the development of TP (28). Therefore, there is a need for focused interventions targeting this group, such as strengthening the entry level, improving routine physical examinations, and increasing the frequency of health surveillance. In addition, our results showed that agricultural work was a high-risk occupation. It may be determined by differences in nutritional status, economic status, and living environment in this specific group (29–32). In the future, emphasis should be placed on assisting traditionally underserved agricultural workers, providing a more comprehensive package, including health checkups and routine health education annually. For some high-risk areas, active surveillance, such as community-based TB screening, should also be employed (33).

As a subtype of TB, patient delay of TP was 5 days shorter than that of TB in China (14). TP generally presented an acute illness, and patients seek medical care earlier (34). Moreover, we found that patient delay of TP was more likely in patients over 65 years of age and retirees. Given that older patients are prone to suffer from different respiratory diseases, diagnosing TP is more difficult than PTB (35). Furthermore, older patients and retirees commonly have an insufficient knowledge of TP, lack care and attention from their family, and face inconvenience of medical appointments, which can be barriers to care (36, 37). In addition, the time interval to the diagnosis of TB in China was 1 (IQR: 0–8) days, which is consistent with our findings (14). In this study, the time interval to the diagnosis of TP was shorter than Peru, India, or Tanzania (38–40). It indicates some success in the prevention and treatment of TP has been achieved in Zhejiang Province, which may be attributed to the relatively developed economy, advanced equipment and instruments, and adequate medical resources. We found that houseworkers or unemployed were at high risk for diagnostic delay. We speculated that this was related to poor compliance with diagnostic testing and treatment in this group. Furthermore, the results showed that the retreatment patients were associated with a high risk of diagnostic delay. Considering the few studies of TP, its potential causes need to be further explored.

The findings of spatial analysis in our research were consistent with the results of spatiotemporal analysis. For patient delay, we found that the hot spots of patient delay were mainly concentrated in the south-central area of Zhejiang Province. The spatiotemporal analysis results showed that the most likely clusters of patient delay were in the midwestern part of Zhejiang Province. For diagnostic delay, this study found that the hot spots of diagnostic delay were mainly concentrated in the east-central and southern areas. The spatiotemporal analysis results showed that the most likely clusters of diagnostic delay were in the southeastern part of Zhejiang Province. These areas have a large agricultural population, relatively low economic status, and limited TP-related knowledge, which are the common risk factors for the development of TP and delay in diagnosis (41). Therefore, it is necessary to reevaluate measures to control the spread of TP in these areas.

Our study had some limitations. First, due to few publications on TP, the comparison was to PTB. Second, although reporting is intended to be uniform, differences in the quality of identification across different regions may have led to inevitable bias. Third, our data were collected from the TBIMS, and some asymptomatic TP cases in the population may not have sought medical services, which may have led to an underestimation of patient and diagnostic delays of TP by our current surveillance system.

## 5 Conclusion

In summary, patient delay of TP in Zhejiang Province was shorter than that for PTB in China, while the diagnostic delay had no difference. Age, city, occupation, and treatment history were related to both types of delays in TP. Some intervention in the central and western regions of Zhejiang Province, such as health education, routine health monitoring, and comprehensive screening, should be initiated to improve the early detection of TP. The allocation of health resources and accessibility of health services should be improved in the central and eastern regions of Zhejiang Province.

## Data Availability

The original contributions presented in the study are included in the article/Supplementary material, further inquiries can be directed to the corresponding authors.
