# Supplementary material for: Spatiotemporal distribution and risk factors for patient and diagnostic delays among groups with tuberculous pleurisy: an analysis of 5-year surveillance data in eastern China
Source: Front Public Health. 2024 Sep 9;12:1461854. doi: 10.3389/fpubh.2024.1461854 (PMC11416949; doi:10.3389/fpubh.2024.1461854)
Supplement: Supplementary file 1 [file Table_1.DOCX]

**Table S1-1** Multifactor logistic regression analysis variable assignment

| **Characteristic** | **assignment** |
| --- | --- |
| Age group (years) | 0 = 0–14;1 = 15-64;2 = 65 and above |
| City | 0=Hangzhou;1=Ningbo;2=Wenzhou;3=Jiaxing;4=Huzhou;5=Shaoxing;6=Jinhua;7=Quzhou;8=Zhoushan;9=Taizhou;10=Lishui |
| Treatment history | 0=Initial treatment;1=Retreatment |

**Table S1-2** Spatial-temporal results of patient delay of TP in Zhejiang Province, 2019-2023

| Cluster type | Cluster period | Location included | Number of counties | Notified cases | Expected cases | LLR | RR | *P* |
| --- | --- | --- | --- | --- | --- | --- | --- | --- |
| Most likely cluster | 2019/4/1 to 2021/9/30 | Jindong, Wuchen, Wuyi, Lanxi, Yongkang, Yiwu, Pujiang, Qujiang, Longyou, Jiande, Liandu, Songyang, Kecheng, Suichang | 14 | 666 | 493.0 | 30.5 | 1.4 | <0.001 |
| Secondary cluster 1 | 2019/7/1 to 2021/12/31 | Huangyan, Linhai, Jiaojiang | 5 | 204 | 135.5 | 15.4 | 1.5 | 0.004 |

**Table S1-3** Spatial-temporal results of diagnosis delay of TP in Zhejiang Province, 2019-2023

| Cluster type | Cluster period | Location included | Number of counties | Notified cases | Expected cases | LLR | RR | *P* |
| --- | --- | --- | --- | --- | --- | --- | --- | --- |
| Most likely cluster | 2019/6/1 to 2021/11/30 | Lucheng, Ouhai, Yongjia, Yueqing Xianju | 5 | 114 | 39.7 | 48.2 | 3.1 | <0.001 |
| Secondary cluster 1 | 2020/9/1 to 2023/2/28 | Yinzhou,Beilun, Fenghua, Dinghai, Zhenhai, Jiangbei, Haishu, Xiangshan Putuo | 9 | 120 | 49.3 | 38.0 | 2.6 | <0.001 |
| Secondary cluster2 | 2021/4/1 to 2023/8/31 | Tiantai | 1 | 29 | 6.8 | 20.1 | 4.4 | <0.001 |
| Secondary cluster3 | 2021/11/1 to 2023/2/28 | Wucheng, Wuyi | 2 | 26 | 6.1 | 18.0 | 4.3 | <0.001 |
| Secondary cluster4 | 2019/12/1 to 2022/5/31 | Anji | 1 | 27 | 8.1 | 13.9 | 3.4 | 0.015 |
